# Biopolymer-Based Mixed Matrix Membranes (MMMs) for CO_2_/CH_4_ Separation: Experimental and Modeling Evaluation

**DOI:** 10.3390/membranes12060561

**Published:** 2022-05-28

**Authors:** Andrea Torre-Celeizabal, Clara Casado-Coterillo, Aurora Garea

**Affiliations:** Department of Chemical and Biomolecular Engineering, Universidad de Cantabria, 39005 Santander, Spain; torrea@unican.es (A.T.-C.); gareaa@unican.es (A.G.)

**Keywords:** chitosan biopolymer-based membranes, CO_2_/CH_4_ separation, experimental and process simulation

## Abstract

Alternative materials are needed to tackle the sustainability of membrane fabrication in light of the circular economy, so that membrane technology keeps playing a role as sustainable technology in CO_2_ separation processes. In this work, chitosan (CS)-based mixed matrix thin layers have been coated onto commercial polyethersulfone (PES) supports. The CS matrix was loaded by non-toxic 1-Ethyl-3-methylimidazolium acetate ionic liquid (IL) and/or laminar nanoporous AM-4 and UZAR-S3 silicates prepared without costly organic surfactants to improve CO_2_ permselectivity and mechanical robustness. The CO_2_/CH_4_ separation behavior of these membranes was evaluated experimentally at different feed gas composition (CO_2_/CH_4_ feed mixture from 20:80 to 70:30%), covering different separation applications associated with this separation. A cross-flow membrane cell model built using Aspen Custom Modeler was used to validate the process performance and relate the membrane properties with the target objectives of CO_2_ and CH_4_ recovery and purity in the permeate and retentate streams, respectively. The purely organic IL-CS and mixed matrix AM-4:IL-CS composite membranes showed the most promising results in terms of CO_2_ and CH_4_ purity and recovery. This is correlated with their higher hydrophilicity and CO_2_ adsorption and lower swelling degree, i.e., mechanical robustness, than UZAR-S3 loaded composite membranes. The purity and recovery of the 10 wt.% AM-4:IL-CS/PES composite membrane were close or even surpassed those of the hydrophobic commercial membrane used as reference. This work provides scope for membranes fabricated from renewable or biodegradable polymers and non-toxic fillers that show at least comparable CO_2_/CH_4_ separation as existing membranes, as well as the simultaneous feedback on membrane development by the simultaneous correlation of the process requirements with the membrane properties to achieve those process targets.

## 1. Introduction

**Figure 1:**
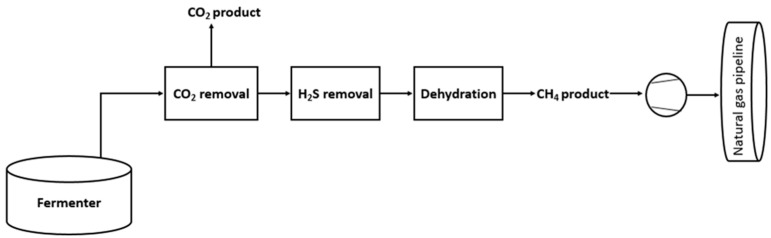
Natural gas upgrading steps adapted from [8].

The concentration of CO_2_ in the atmosphere is in continuous growth, by the last century it has increased from 275 to 418 ppm, which has already produced irreversible increases in global temperatures and the concentration of this component is expected to continue to increase over time [1]. Energy production is necessary to face the demand of the growing world population [2]. To face this problem, one possibility is the recovery of biogas to obtain energy [3]. Methane, upgraded from biogas, can be used for heat and electricity production or as biofuel for vehicles to reduce environmental emissions and the use of fossil fuels [4]. Biogas is produced from the methanation of biomass and organic wastes from sewage sludge anaerobic digestion, commercial composting, landfills, biomass gasification, animal, and food waste [5]. Biogas usually contains from 55% to 65% methane, from 35% to 45% carbon dioxide and less than a 1% nitrogen and other traces of sulfur compounds, siloxanes, and aromatic compounds, which can contribute to stratospheric ozone depletion, greenhouse effect, and reduction of the quality of air, as well as cause corrosion and maintenance problems in the gas pipelines [6]. Carbon dioxide (CO_2_) is the other major component of biogas that decreases the density and calorific value of the biogas, thus the importance of separating CH_4_ from CO_2_ to increase the value and use of biogas as biomethane [5]. Figure 1 shows the different steps in biogas upgrading, with CO_2_ removal being the most important cleaning step. CO_2_/CH_4_ is still a challenging separation due to the variability of the biogas composition, which mainly depends on the source and seasonal conditions as well as the huge amount of water leaving the fermenter that makes necessary a dehydration step in conventional processes, such as chemical absorption [7].

In this scenario, membrane technology has been selected as one of the efficient technologies to reduce CO_2_ emissions in the atmosphere by separation/capture processes from the flue gas, such as natural gas and synthesis gas, achieving sustainable development goals with the creation and utilization of sustainable energy sources [9,10]

The most important properties of membranes to perform this separation are: (1) high CO_2_ permeability, (2) high CO_2_/CH_4_ selectivity, (3) stability of the material, (4) good resistance to aging and plasticization, (5) sustainability and reproducibility of the manufacturing to levelize the fabrication cost upon up-scaling [11]. The most matured membranes for CO_2_ separation are based on polymeric materials. Some polymers investigated in literature for CO_2_ separation are polysulfone (PSf) [12], polyimide (PI) [13], polyetherimide (PEI) [14,15], polycarbonate (PC) [16,17], polyethersulfone (PES) [18], but the number of CO_2_ selective polymer membranes close to commercialization are still limited: Polaris^®^, Polyactive™ and facilitated transport membranes [19]. Even those commercial membranes experience some disadvantages, such as the uncertainty of their performance regarding the presence of impurities, such as water and organic vapors and doubts on the thermal, mechanical, and chemical resistance, as well as the costly fabrication methods [20,21,22].

Although membrane separation processes are considered eco-friendly technologies, there is still the necessity to improve the sustainability of the membranes themselves using renewable and environmentally friendly materials in their fabrication. Biopolymers can be obtained from various sources and have recently been considered as potential materials for replacing different chemically synthetized polymers used in membrane fabrication due to their properties, such as biocompatibility, biodegradability, compostability and environmental sustainability [11,23,24]. Cellulose acetate (CA) was the first biopolymer to be used for membrane separation because of its properties, such as easy processability, versatility and low environmental footprint. The main disadvantage is that this biopolymer is susceptible to plasticization as a consequence of CO_2_ sorption, generally at high pressure [11]. Moghadassi et al. functionalized a CA membrane using multiwalled carbon nanotubes, providing an increase in perm-selectivity compared to pristine CA membranes. Moreover, the addition of organic additives, such as polyethylene glycol (PEG), improved the flexibility of the membrane, implying an increase in permeability and aging resistance [25]. Mubashir et al. incorporated NH_2_-MIL-53(Al) into a CA matrix, increasing CO_2_ permeability from 15.5 Barrer to 52.6 Barrer and CO_2_/CH_4_ selectivity from 10.7 to 28.7 [26]. Other bio-based polymer materials are becoming more popular in membrane processes (Table A1 in Appendix A). PVA is a synthetic biodegradable, low-cost and non-toxic polymer with high hydrophilicity and good barrier properties, which is easily blended with other polymers to reduce the crystalline fraction and increase membrane performance [27,28]. Polyvinyl alcohol PVA was blended by polyvinyl amine (PVAm) to provide the selective layer of the membrane with a facilitated transport mechanism and increase the CO_2_/CH_4_ separation performance regardless of the relative humidity of the fuel gas [29]. PVAm:PVA blend membranes loaded with carbon nanotubes (CNTs) showed improvements on the durability, mechanical resistance, CO_2_ permeability and CO_2_/CH_4_ selectivity compared to the pure polymeric membrane [30]. Jahan et al. coated PVA blended with crystalline nanocellulose (CNC) on a PSf support for biogas upgrading. They observed that the addition of CNC influenced the water swelling degree, crystallinity, and thickness of the resulting membranes, facilitating the transport of CO_2_ compared with the pristine PVAm:PVA membrane [31]. Another biopolymer, polyurethane (PU), obtained from polyols in plant oils has been recently reported for gas separation on account of properties of hydrophobicity, low rigidity, stable barrier properties, good mechanical resistance, and water-vapor permeability. Again, the addition of nanoparticles can improve the gas separation performance by increasing free volume as well as thermal and mechanical properties. Molki et al. observed that NiO NPs as filler in PU increased CO_2_ permeability while decreasing CH_4_ permeability, which is a larger molecule [32]. Ghadimi et al. [33] added PEG to polyurethane and they crosslinked prepared membranes using a methoxysilane-functionalized ionic liquid (Si-IL). The IL used was made from the BF_4_^-^ anion with high affinity for CO_2_ and the siloxane loading up to 10wt.% increased both the CO_2_ permeability and selectivity of the PEG/PU hybrid matrix. The inorganic siloxane domains incorporated into polymeric matrices increases the fractional free volume of the membrane matrix. Pyridinium based Ils have been reported to increase the mechanical stability of CA based membranes in CO_2_ separations [34]. Sodeifian et al. [35] used SAPO-34 zeolites as nanoporous fillers in PU MMMs. The differences in kinetic diameter, condensability, and interaction with the polar groups of the polymer and then porosity of the zeolite caused larger increases in the CO_2_ permeation rate than that of CH_4_. Although these membranes provided a 4.45% and 18.24% reduction of CO_2_ and CH_4_ permeability, respectively, CO_2_/CH_4_ selectivity increased about 14.43%_._ Layered materials have been reviewed to improve gas permeation properties of PES membranes using ionic liquid as a binder between the filler and the continuous matrix [18].

Chitosan (CS) is a non-toxic biopolymer from renewable sources, with low cost, biocompatibility, and high hydrophilicity [36]. CS is a semi-crystalline polymer that contains one hydroxyl group and one amine group in its structure, which are responsible for the facilitated transport of CO_2_ across this material [37]. The main properties of biopolymers include high hydrophilicity, low mechanical resistance, variable pore size and membrane morphology [38]. Further, the removal of the impurities in biogas, in particular CO_2_, may reduce mass transfer limitations due to the hydrophilic character of the membrane [39]. Fewer studies applied CS-based membranes to CO_2_/CH_4_ than CO_2_/N_2_ separation. Jomekian et al. prepared CS modified g-C_3_N_4_ using PES as porous support and ZIF-8 as filler to increase the performance of the membrane [40]. They observed an increase in selectivity in pure gas experiments when CS-modified was mixed with ZIF-8 compared to ZIF-8 pristine membrane. However, mixed gas experiments showed the same trend, but lower separation factor, due to the competing effects in the penetration of the gases through the membrane [40]. In previous works, 1-ethyl-3-methylimidazolium acetate, [emim][Ac] IL was observed to increase the thermal and mechanical behavior of CS membranes for CO_2_/N_2_ separation [41]. Mixed-matrix membranes (MMMs) are a well-known route to enhance the properties of polymeric membranes by incorporating an inorganic material in the form of micro- or nanoparticles (filler) into the polymeric matrix (continuous phase) [42]. The addition of ETS.10 titanosilicate [43] and nano-sized ZIF-8 and HKUST-1 particles also improved selectivity and permeability in dense self-standing films on account of the compatibility between the filler and the continuous IL-CS matrix, which we have also studied by Hansen solubility parameters [44]. The successful development of MMMs depends on the proper selection of the polymer that forms the matrix and the inorganic filler, also on the elimination of interfacial defects between both phases. Another crucial factor is to control filler loading, shape, and size to achieve the best performance [45]. These MMMs can be coated into porous supports that provide mechanical resistance, allowing for the reduction of the thickness of the selective layer and thus increasing membrane productivity without diminishing the selectivity of the self-standing material [46]. The functional groups of the biopolymers and fillers have proven to be useful robust CO_2_ carriers in solid facilitated membranes [22]. IL-CS/PES composite membranes using the same IL and HKUST-1 nanoparticles as filler of the CS matrix over a flat polyethersulfone (PES) support [36] or hollow fibers. Furthermore, promising results were obtained for these HKUST-1-CS:IL composite membranes, at a CO_2_/CH_4_ (50:50v%) feed mixture composition, as selectivity increased from 12 to 30 in wet conditions while CO_2_ permeability was maintained at a value of 400 GPU regardless the HKUST-1 loading [47]. Layered AM4-4 and UZAR-S3 silicate materials have proved good interfacial contact with CS-based matrices on account of the compatible ion exchange capacity of both and the high aspect ratio of the 2D nanosheets [48,49] that promotes the CO_2_ adsorption capacity of the delaminated AM-4 in comparison with the layered precursor [50] and the thickness of the nanosheet exfoliated layers of UZAR-S3 [51].

This is the reason why these fillers were selected in this study for the preparation of MMM composite membranes based on IL-CS continuous matrix for the selective layer and different fillers ([emim][Ac] IL [41], layered AM-4 titanosilicate [50], layered UZAR-S3 stannosilicate [51] and HKUST-1 metal organic framework (MOF) [44]). The membranes were coated on PES support in order to evaluate the potential to simultaneously increase the permeability, selectivity, and stability of the membranes in a simple way. The performance of the membranes in single gas and CO_2_/CH_4_ mixture separation was experimentally evaluated at different feed gas mixture compositions and analyzed and compared with other biopolymer-based membranes in the literature using Aspen Custom Modeler. A cross-flow model of membrane cell was validated for the process simulation using a well-known commercial membrane in CO_2_ separation applications, by testing different feed gas composition covering different separation scenarios associated with CO_2_/CH_4_ separation, such as natural gas sweetening, biogas upgrading, and enhanced oil recovery where the gas is richer in CO_2_. A sensitivity analysis as a function of the stage-cut as a key process variable was performed and compared with other referenced biopolymer-based membranes.

## 2. Materials and Methods

### 2.1. Membrane Preparation

The membranes used for this study are flat-sheet composite membranes synthetized in our laboratory. Commercial PES membranes with a 0.1 μm pore size and a thickness of 132 μm have been used as support for the composite membranes. The top layer coated is made of the chitosan biopolymer (CS) purchased from Sigma Aldrich (Spain) with a few drops (5 wt.% to total solid content) of 1-Ethyl-3-methylimidazolium acetate [emim][Ac] IL (Sigma Aldrich, Spain), after results obtained previously in our research group [41,46]. In brief, prior to the coating of the hydrophilic biopolymer layer, the surface of the PES support was coated by a hydrophobic solution of PDMS/hexane or trimesoyl chloride (TMC)/Hexane 0.1wt.% that prevented cluster or crystallization on the surface pores of the support and allowed a homogeneous coating layer thickness. The IL-CS matrix was loaded by different types of nanometric fillers prepared by a method reported elsewhere (AM-4 [52], UZAR-S3 [53] and HKUST [44]) that have been added to the polymeric membrane in a 10 wt.% of the total mass of the polymer matrix [49]. For the synthesis of the two nanoporous silicates, sodium silicate (Na_2_SiO_3_), sodium hydroxide (NaOH), Anatase (TiO_2_), tin(II) chloride di-hydrated (SnCl_2_ 2H_2_O) and copper chloride·5 H_2_O that were acquired from Aldrich (Spain) were used. Different MMMs were prepared by solution casting at room temperature [44].

The thickness of the prepared membranes was measured by means of a digital Mitutoyo digimatic micrometer (IP 65) with an accuracy of 0.001 mm. The average thickness of the selective layer of the membranes was 43.63 ± 9.27 µm (more details in Table A1, Appendix A). The differences in thickness between the hydrated and dry film allows for monitoring the degree of swelling and the mechanical robustness of the membrane before and after gas separation runs [43]. The experimental density of the membranes (*ρ_m_*) can be measured gravimetrically from the electronically measured weight of the dry film and the volume calculated from the dry thickness at room temperature (20 °C).

The water uptake of the IL-CS based membranes was measured after the activation step in NaOH 1 M and rinsing in DI water. The membranes were immersed in DI water for at least 25 h. The wet weight was obtained by quickly blotting the membrane on tissue paper to remove the excess water. The total water uptake was calculated as:

(1) $$WU\left(\%\right)=\frac{W_{wet}-W_{dry}}{W_{dry}}\times 100$$

where *W_dry_* is the dry weight of the membrane, and *W_wet_* the weight of the swelling membrane, both in grams. The porosity of the membrane has been calculated as in previous works, from the volume occupied by water and the volume of the membrane, considering the water density at 20 °C (0.998 g/cm^3^) and the density of the membrane in dry state [44]. The void fraction, is thus calculated as:

(2) $$\varphi_{v}=\left(\;\frac{W_{wet}-W_{dry}}{\rho_{water}}\right)+\frac{W_{dry}}{\rho_{m}}$$

The water content of the membranes was measured before and after every experimental run (all feed concentrations) until constant values, in order to verify that the gas permeation runs were conducted under constant humidity.

ATR-FTIR spectroscopy was performed using a Perkin Elmer spectrometer over 4 scans with a wave number resolution of 4 cm^−1^ in the range 400–4000 cm^−1^.

### 2.2. Gas Separation Experiments

**Figure 2:**
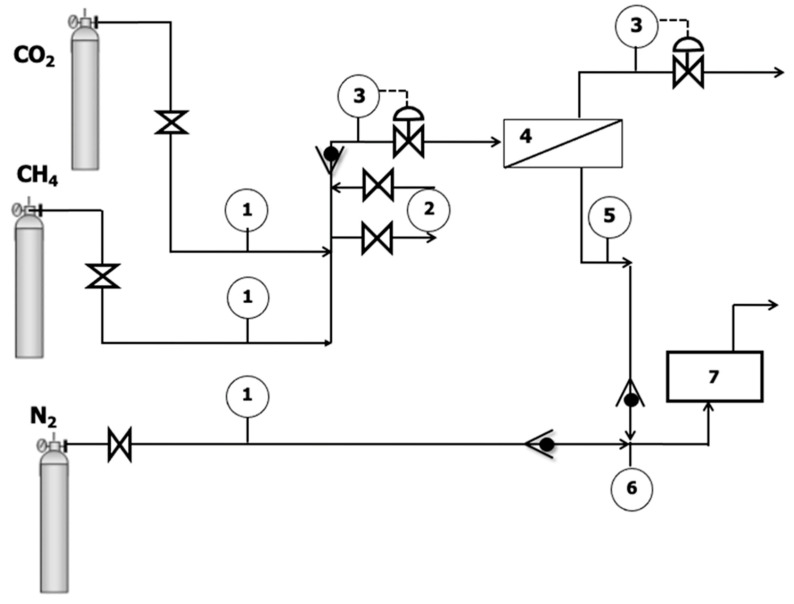
Experimental setup. (1) Mass flow controller, (2) Water bubbler, (3) Feed and retentate pressure regulator, (4) Membrane module, (5) Permeate pressure indicator, (6) Permeate flowmeter, (7) Gas analyzer.

The MMM composite membranes provide an effective membrane area of 15.6 cm^2^. For the characterization of these membranes, they were placed in a stainless-steel module, which consists of two stainless steel pieces with a cavity where the membrane is placed using a 316LSS microporous disk support with a pore size of 20 μm and sealed by Viton Rings. Pure gas permeance of CH_4_ and CO_2_, in this order, was obtained using the home-made separation plant represented in Figure 2. The feed flow rate was set to 50 mL/min using mass flow controllers (KOFLOC 8500, Sequopro S.L., Madrid, Spain). The permeate flow rate was measured using a bubble flow meter at the exit of the membrane module. Feed pressure was set at 4 bar. Permeation experiments were run for 2 h for each pure gas, in order to ensure conditioning and steady state.

To carry on the validation of the model, different feed mixtures of CO_2_/CH_4_ were introduced to the system in relations of 35:65, 50:50, 20:80 and 70:30 v%, respectively, of each gas, being the possible biogas composition depending on the source. The permeate flow rate was measured at the exit of the entire system in the same way as pure gases, and to establish the composition of the permeate, a gas analyzer (BIOGAS5000, Geotech, Tamarac, FL, USA) was used. The experimental results of the permeate gas stream thus obtained for the PDMS commercial membrane were compared with the simulated results to validate the model described below.

The permeance of the gas *I* in units of GPU (1 GPU = 10^−6^ cm^3^ (STP) cm^−2^ s^−1^ cmHg^−1^) is defined as the pressure-normalized flux of a gas through a membrane:

(3) $${\left(\frac{P}{l}\right)}_{i}=\frac{Q_{p}}{\left(p_{r}-p_{p}\right)A}\times \;10^{6}$$

where *P* is the intrinsic permeability of the selective membrane layer, in Barrer (1 barrer = 10^−10^ cm^3^ (STP) cm^−1^ s^−1^ cmHg^−1^); *p_r_* and *p_p_* is the retentate and permeate pressure (bar), respectively; *A* is the effective area of the membrane (cm^2^); *l* is the selective layer thickness for the separation; *Q_p_* is the permeate flow rate (cm^3^ (STP) s^−1^) at measurement pressure and temperature conditions.

The selectivity of the membrane is defined by the ratio between the permeability of both pure gases across the membrane:

(4) $$\alpha_{ij}=\frac{P_{i}}{P_{j}}$$

### 2.3. Process Simulation: Membrane Unit Model

**Figure 3:**
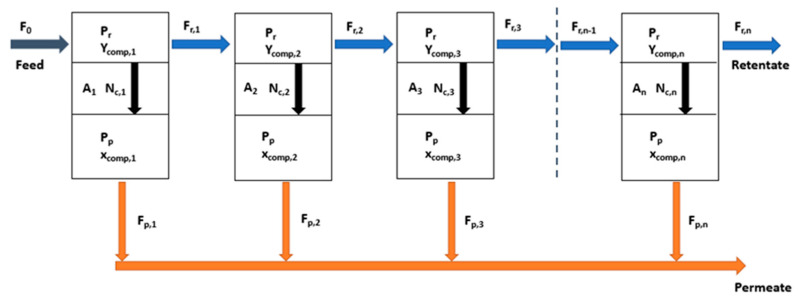
Diagram of the crossflow membrane model.

A crossflow membrane model was built using Aspen Custom Modeler. For membrane modeling a tank in series model was applied where the membrane unit is divided in k number of equal sized uniform cells (being *k* variable from 1 to *n*), where the permeate of each cell is recovered and mixed with the rest of the permeate streams, the retentate of each cell being the feed for the next one, as represented in Figure 3 [52].

The main assumptions for this model in each cell are the following [53]:

- Ideal gas behavior.
- Isothermal and constant permeance.
- The pressure drop is negligible at each side of the membrane.
- The effect of concentration polarization is negligible.
- The permeance depends on the feed conditions and can be estimated based on correlations dependent on conditions, including pressure, flowrate, and composition.

According to the previous assumptions, steady state material balances were used to describe the changes in gas composition and flowrates at both sides of the membrane, whose expressions are shown in Equations (5)–(7) as follows.

For any *k* cell from 1 to *n*:

(5) $$F_{r,k-1}=F_{r,k}+F_{p,k}$$

(6) $$F_{r,k-1}\;y_{comp,k-1}=F_{r,k}\;y_{comp,k}+F_{p,k}x_{comp,k}\;$$

(7) $$N_{comp,k}=F_{p,k}\;x_{comp,k}\;$$

In these equations, *F_r,k_* and *F_p,k_* are the total molar flowrate (kmol h^−1^) of retentate and permeate leaving each cell respectively, *x_comp,k_* and *y_comp,k_* are the molar fraction of each component in the mixture present in the permeate and retentate streams and *N_comp,k_* is the molar flowrate of each component permeating though the membrane cell *k* (kmol h^−1^) [53].

To describe the transport mechanism across the membrane the solution-diffusion model is used. In this model, the partial pressure difference across the membrane is the driving force of permeation [54]:

(8) $$N_{comp,k}=A_{k}\;P_{comp}\;\left(p_{r}\;y_{comp,k}-p_{p}\;x_{comp,k}\;\right)$$

This is described by *P_comp_* being the permeance for each component across the membrane converted to molar basis (kmol h^−1^ bar^−1^ m^−2^, calculated from the experimentally obtained permeance in m^3^ (STP) h^−1^ bar^−1^ m^−2^ units), *A*_k_ is the membrane area of the cell *k*, and *p_r_* and *p_p_* are the pressure on the retentate and permeate sides of the membrane, respectively.

At the *n*th cell, outlet of the membrane, the retentate molar flowrate and the molar fraction of each component in this stream are the calculated for *k* = *n*, while the permeate molar flowrate of the outlet stream is obtained as the sum from *k* = 1 to *n,* and the corresponding molar fraction of components as follows:

(9) $$F_{p,n}=\sum_{k=1,n}\left(F_{p,k}\right)$$

(10) $$F_{p,n}\cdot x_{comp,n}=\sum_{k=1,n}\left(F_{p,k}\;x_{comp,k}\right)$$

The stage-cut is the ratio of the permeate flowrate to the feed flowrate, as shown in Equation (9) [55]:

(11) $$\theta =F_{p}/F_{f}$$

To compare the separation performance of different membranes, two parameters are used: the purity and recovery of each component across the membrane, in:

(12) $${\mathrm{Purity}\;}_{comp}\left(\%\right)=100\times \frac{F_{comp,out}}{F_{out}}=100\times \frac{F_{comp,out}y_{comp,out}}{F_{out}}=100\times y_{comp,out}$$

(13) $${\mathrm{Recovery}}_{comp}\left(\%\right)=100\times \frac{F_{comp,out\;}}{F_{comp,in}}=100\times \frac{F_{out}y_{comp,out}}{F_{in}x_{comp,in}}$$

Both parameters are considered as the most important parameters to determine the effectiveness of the simulated membranes and all the specifications are focused on their values so that the optimization of the process aims at obtaining the maximum purities and recoveries of each component in the permeate for the most permeable one and on the retentate for the other. In response to the difficulty of maximizing both process parameters at the same time., a multi-objective problem would be proposed for establishing more global targets [56]. Another aspect that should be fixed before undertaking the simulation is the number of cells, *n*, required to achieve a determined objective. The magnitude of this process parameter determines the precision of the results obtained. In this work, we selected a value of *n* = 100 as a compromise between the numerical discretization and the model precision [56].

## 3. Results

### 3.1. Pure Gas Permeation Experiments

**Figure 4:**
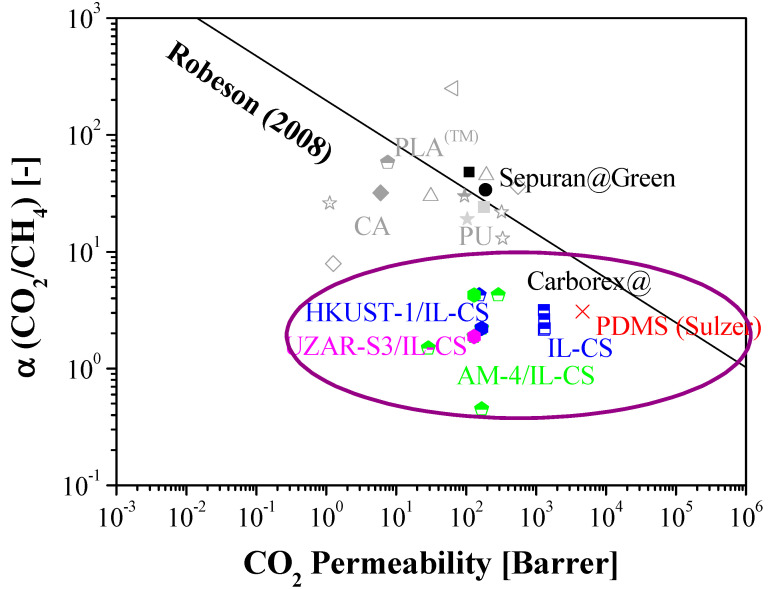
CO_2_/CH_4_ selectivity and CO_2_ permeability of biopolymer-based mixed matrix and composite membranes against the 2008 Robeson upper bound [57]. Commercial membranes used for biogas separation are represented in grey and black for comparison. The values obtained in the single gas permeation experiments in this work are contained within the circle.

The results of single CO_2_ and CH_4_ permeation are plotted against the 2008 Robeson upper bound commonly used for comparing permselective membrane materials for a specific gas pair separation target, since it describes the trade-off of existing polymer materials to perform a certain gas pair separation [57]. Advancements on highly permeable advanced polymers and complex configurations, such as facilitated transport membranes, composite flat, and hollow fiber membranes, have led to revisions of these upper bound [58,59]. Several biopolymer MMM and composite membranes reported in literature [11] regarding this target separation are also included in Figure 4. The values are collected in Table A1 (Appendix A). The data points of the mixed matrix composite flat membranes evaluated in this work are encircled for comparison with literature.

A large number of fillers have been used in mixed matrix membranes for CO_2_ separation. It is very important that the filler should have good compatibility with the polymer matrix to increase CO_2_ separation performance, to avoid the formation of interfacial voids between the dispersed phase (fillers) and continuous matrices that imply a reduction in the CO_2_ permselectivity of the membrane by allowing the transport of other species present in the feed though the voids [24]. Mixed matrix composite flat membranes made of thin layers of IL-CS loaded with nano-HKUST-1 observed an increase in CO_2_ permeability and CO_2_/CH_4_ selectivity, attributed to the compatibility of the components in the MMM [44] that can be transferred to composite membranes when this MMM is coated on a compatible support [46]. This increase was even more apparent in wet conditions because of the high hydrophilicity of these material. The addition of two-dimensional fillers with higher aspect area/volume ratio as layered AM-4 [51] and UZAR-S3 [52] stannosilicate also seemed promising, although the permselectivity ratio is still low in thin composite form. This is attributed to the different hydrophilicity character of the material components [29,60]. The water uptake (Equation (1)) and dimensional swelling degree are calculated after Equations (1) and (2), respectively, and collected in Table 1. The water uptake is a measure of the hydrophilicity of the membrane and the dimensional swelling of the mechanical robustness upon performance. The porosity of the composite membranes studied in this work had an average value of 34 ± 2%. The porosity (or void fraction) for the selective layer was 6 ± 2%. The average thickness of the selective layer was lower than 25 µm in the dry state. These results may support the evidence regarding the role of hydrophilicity on the CO_2_ separation performance [61].

**Figure 5:**
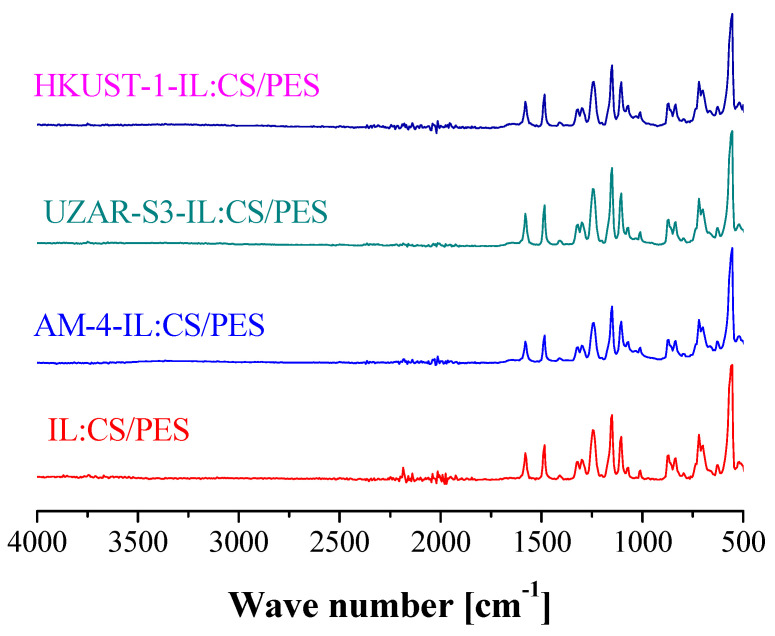
ATR-FTIR spectra of the IL:CS/PES based composite membranes as a function of MMM layer composition.

The ATR-FTIR spectra of dry IL-CS/PES composite membranes are shown in Figure 5. The analyses were performed after the gas permeation runs and drying to constant weight in order to avoid the masking of the intrinsic peaks of the selective layer materials by the abundant presence of hydroxyl groups due to the water adsorbed in them [62]. The intensity appears to be independent of the type of filler in the IL-CS matrix. The bands of the MMM layer do not shift from the IL-CS spectra, thus implying that the interaction between the filler and the continuous matrix is good and homogeneously dispersed as studied in previous works upon the self-standing films [63].

### 3.2. Process Simulation and Sensitivity Analyses

#### 3.2.1. Model Validation

**Figure 6:**
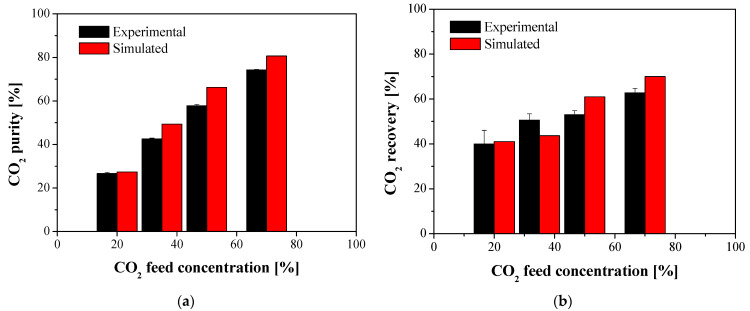
Experimental and simulated CO_2_ permeate purity (**a**) and recovery (**b**) as a function of CO_2_ feed concentrations for a PDMS commercial membrane. (Temperature = 20 °C, feed pressure = 4 bar).

For the membrane cell modeling, the experimental process conditions as well as the membrane parameters, in terms of selectivity and permeability, were required for the simulation task in order to evaluate the process performance in terms of purity and recovery of CO_2_ at the permeate side, which were used for the model validation, and the corresponding parameters of CH_4_ in the retentate side. This validation was made with a PDMS commercial membrane, which provided a single CO_2_ gas permeance value of 369.29 GPU and a CO_2_/CH_4_ selectivity of 3.11 (Equation (4)). The experimental results obtained for the separation of CO_2_/CH_4_ gas mixtures using a commercial PDMS membrane were used to validate the model Equations (12) and (13), for the target objectives of purity and recovery, respectively. The simulated values of the CO_2_ permeate purity and recovery were determined by introducing the experimental CO_2_ permeance and CO_2_/CH_4_ selectivity at each feed composition, as well as the experimental stage-cut, into the developed model. Figure 6 compares those CO_2_ permeate purity and recovery experimental (black) and simulated (red) values (%) as a function of the feed gas mixture. The model is validated by the experimental results of the separation of CO_2_/CH_4_ mixtures with the commercial PDMS membrane.

The deviation between the experimental and simulated results was estimated as an absolute relative error, AARE, using Equation (14). The values obtained for the CO_2_ and CH_4_ permeate purity and recovery using the commercial PDMS membrane as reference, are collected in Table A2 and Table A3 and by applying material balances, Table A4 and Table A5 represent the retentate purity and recovery, respectively (Appendix A). Except for the experiment of CO_2_/CH_4_ 30:70 v% feed, which corresponded to AARE values for the CH_4_ component around 25%, the AARE values obtained for the PDMS reference membrane validate the model within acceptable range (below 20% and significantly lower values):

(14) $$\mathrm{AARE}=100\times \left|\frac{Simulated\;Value-Experimental\;Value}{Experimental\;Value}\right|,$$

Considering the results obtained for the experimental and the simulation studies, if we consider the existence of an experimental error when obtaining the mean value of the two parameters represented with their standard deviation, it can be stated that the fit of the model is adequate to describe the performance of the membranes characterized by its permeability and selectivity.

#### 3.2.2. Influence of Feed Concentration

**Figure 7:**
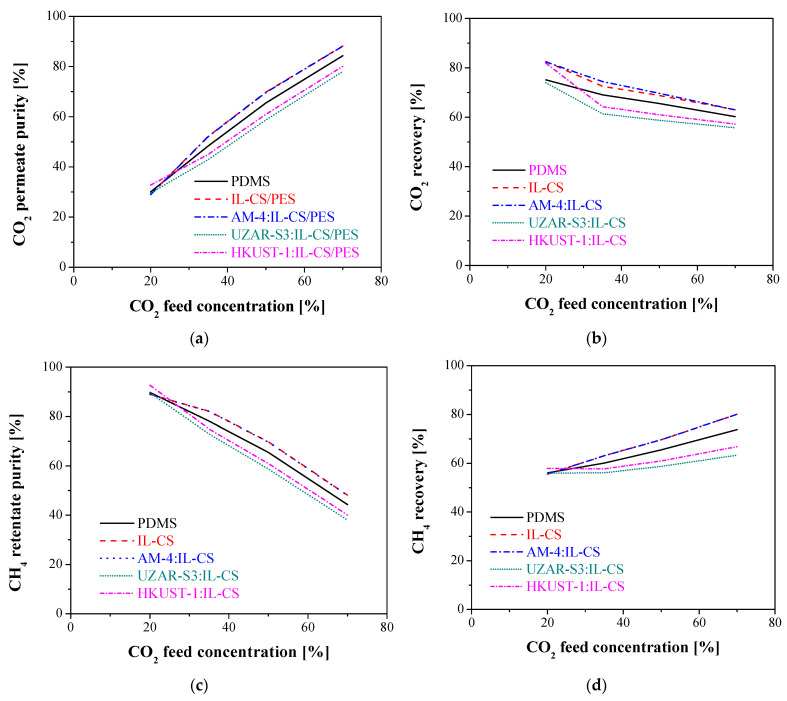
Influence of the CO_2_ feed concentration in (**a**) CO_2_ purity in the permeate, (**b**) CO_2_ recovery, (**c**) CH_4_ purity in the retentate, and (**d**) CH_4_ recovery for all the membranes tested in this work at the experimental stage-cut of 0.5 and a feed pressure of 4 bar.

The experimental results were used to validate the developed mathematical model. The CO_2_/CH_4_ gas mixture separation of the chitosan based composite membranes was thus compared with the reference commercial PDMS membrane. Figure 7 shows the results of the simulations of the permeate purity and recovery selected as target objective variables as a function of CO_2_ concentration in the feed. The stage-cut and feed pressure considered for the modelling equations were 0.5 and 4 bar, respectively, in agreement with the experimentally obtained values. The CO_2_ purity in the permeate increases while the recovery decreases with increasing CO_2_ concentration in the feed, which indicates that some CO_2_ goes to the retentate, decreasing the recovery of the CH_4_ in the retentate [56]. All the membranes observe a similar trend and IL-CS and AM-4:IL-CS composite membranes provide the largest values of CO_2_ and CH_4_ purity, closely followed by the commercial PDMS membrane. The purity values of IL-CS and AM-4:IL-CS composite membranes reached a CO_2_ purity of 88% at high CO_2_ concentration in the feed and a maximum recovery of around 82% against the 84% CO_2_ purity and maximum recovery of 75% from simulation with the commercial membrane. The CO_2_ purity and recovery with UZAR-S3:IL-CS and HKUST-1:IL-CS composite membranes are 80% and 75%, respectively. As expected, the performance of CH_4_ purity and recovery is the opposite with increasing CO_2_ concentration in the feed. CH_4_ purity is reduced from 88.9% to 48% for the CS-IL and AM-4: IL-CS composite membranes, and from 89% to 40% for the other IL-CS-based composite membranes. The recovery of CH_4_ increases 10% for the UZAR-S3- and HKUST-1: IL-CS membranes, against 20% for the PDMS commercial membrane, whereas AM-4:IL-CS composite membrane observed a CH_4_ recovery up to 30%. The best behavior in CO_2_/CH_4_ separation and recovery is thus related to the hydrophilic character of both the CS-based matrix and the AM-4 filler, since hydrophilicity has been observed to facilitate the transport through water-swollen composite membranes [29]. The AM-4 filler is also more easily dispersed than UZAR-S3 and HKUST-1 in the CS matrix [46,49] and provides additional CO_2_ adsorptive properties [50] to the membrane when embedded in the IL-CS matrix of the selective layer of the membrane. These results give scope for the possibility of substitution of PDMS by renewable materials.

According to these results, variations in the feed concentration have a stronger impact on the purity than in the recovery of both components in the outlet streams.

#### 3.2.3. Influence of the Stage-Cut

**Figure 8:**
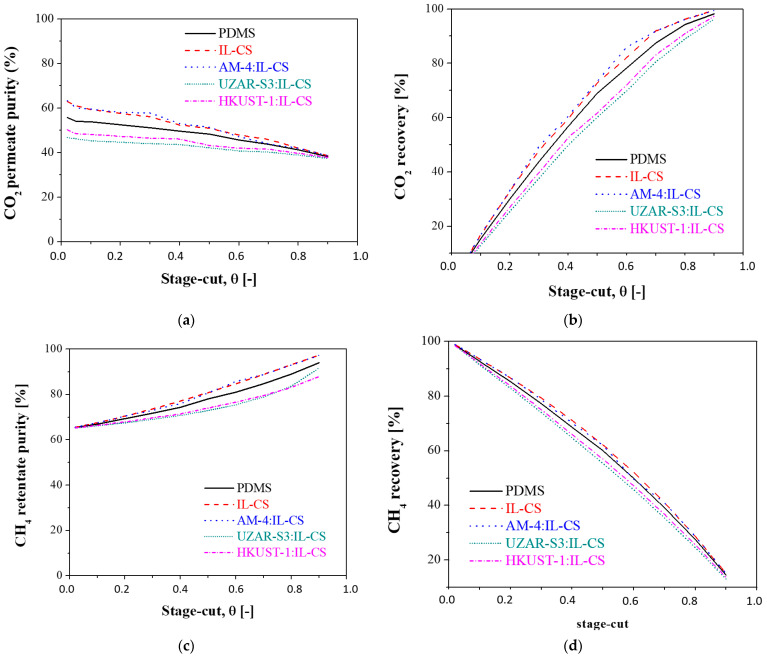
Influence of the stage-cut for the membranes tested in this work on: (**a**) the CO_2_ purity in the permeate, (**b**) the CO_2_ recovery in the permeate, (**c**) the CH_4_ purity in the retentate, and (**d**) the CH_4_ recovery in the retentate. Feed concentration: 35% CO_2_: 65% CH_4_, feed pressure: 4 bar.

A sensitivity analysis to study the capacity of the synthesized membranes via the stage-cut variable is presented below, at a fixed feed concentration of 65% of CH_4_, 35% of CO_2_, simulating the base raw biogas concentration without the trace components (hydrogen sulfide, water vapor, ammonia, and siloxane) that may be present depending on the types of feedstock and digestion process [63]. Different values of stage-cut, ranging from 0.1 to 0.9, were considered to calculate the evolution of purity and recovery of CH_4_ in the retentate and CO_2_ in the permeate, which are plotted in Figure 8.

In Figure 8, an increase in the CH_4_ purity in the retentate and the CO_2_ recovery in the permeate are observed with increasing stage-cut. While a high value of stage-cut is needed to attain a CO_2_ recovery close to 100%, the CO_2_ purity of all the membranes tested in this work was below 65% in all the range of stage-cut studied. This confirms the compromise between these target objective parameters. The increase in CH_4_ purity in the retentate with increasing stage-cut is lower than that of CO_2_ purity, from an initial 66% to a final 94% for the commercial PDMS, 91.7% for the UZAR-S3:IL-CS membrane, and 87.9% for the HKUST-1:IL-CS membranes. Again, IL-CS and AM-4:IL-CS membranes reached the highest CH_4_ purities of 97.2%. The reduction in CO_2_ purity with increasing stage-cut ratio is related to methane losses in the permeate stream due to the higher amount of gas passing through the membrane. This explains the reduction in CH_4_ recovery in the retentate, going from 92.8%, 93.6%, 93.5%, 91.5% and 91.9%, to 14.5%, 15%, 15%, 12.9% and 13.5% for PDMS, IL-CS, AM-4:IL-CS, UZAR-S3:IL-CS and HKUST-1:IL-CS composite membranes, respectively, when stage-cut increases from 0.1 to 0.9. The CO_2_ recovery would be drastically increased from around 15% to 98.3% for PDMS, 99.5% for the IL-CS and AM-4:IL-CS, 96.2% and 97.4% for the UZAR-S3:IL-CS and HKUST-1:IL-CS composite membranes, respectively. These results give evidence again of the significance of hydrophilicity and CO_2_ adsorptive properties of the components of the top layer of the composite membrane upon CO_2_ separation [64].

**Figure 9:**
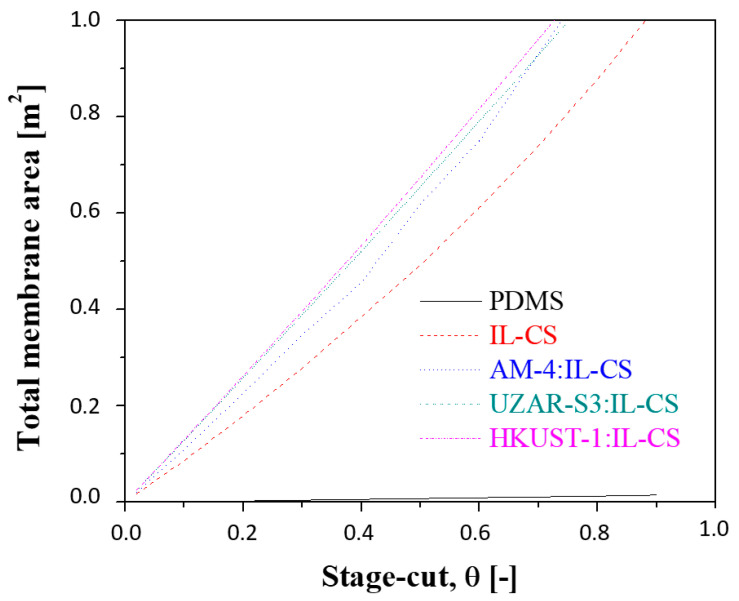
Evolution of the required membrane area as a function of stage-cut. Experimental feed flow rate: 50 mL/min, feed pressure: 4 bar. Feed concentration: 35% CO_2_: 65% CH_4_.

The relationship between the stage-cut and the required total membrane area is plotted in Figure 9. As expected, higher stage-cut values require a larger membrane area. The membrane area required for the PDMS commercial membrane is included for comparison, although the value is ten-fold less than the IL-CS-based composite membranes because of the higher experimental permeation rate.

### 3.3. Comparison with Literature

**Figure 10:**
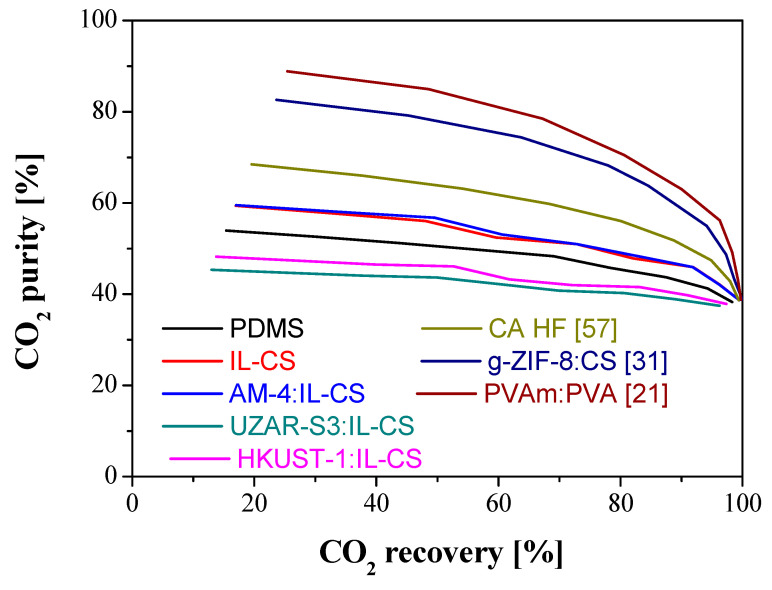
Comparison of the results of CO_2_ purity versus CO_2_ recovery in the permeate for the membranes prepared in this study with the three biopolymer-based membranes selected from the literature. Feed concentration: 35% CO_2_: 65% CH_4_. Feed pressure: 4 bar. Feed flow rate: 50 mL/min.

**Figure 11:**
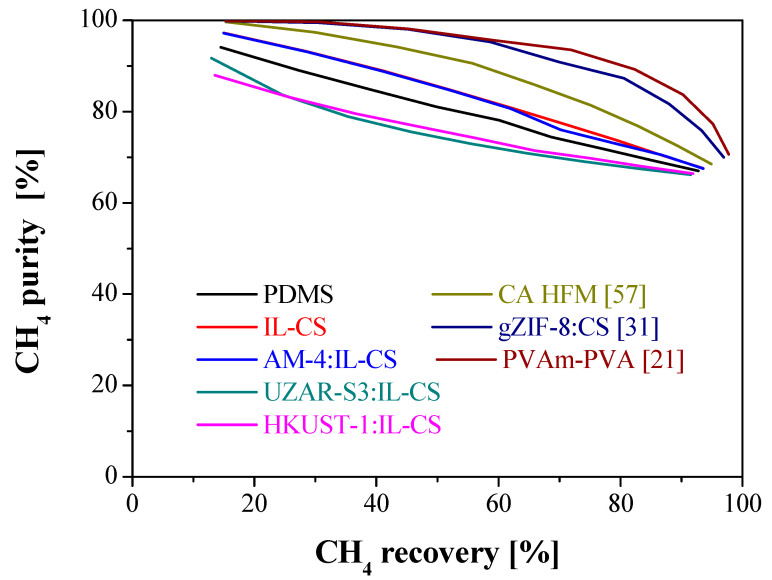
Comparison of the results of CH_4_ purity versus CH_4_ recovery in the retentate for the prepared membranes in this study with three selected membranes based on biopolymers from the literature. Feed concentration: 35% CO_2_: 65% CH_4_. Feed pressure: 4 bar. Feed flow rate: 50 mL/min.

Membranes prepared with three other type of biopolymers were selected from the literature to be compared with the membranes presented in this study regarding the target objectives and CO_2_/CH_4_ separation performance. These membranes were selected on account of the different values of CO_2_ permeance and CO_2_/CH_4_ selectivity to check the applicability of the model to membranes in a wider range of membrane properties. These membranes are a CA hollow fiber membrane with a CO_2_ permeance of 248 GPU and a CO_2_/CH_4_ selectivity of 7.9 [65], a ZIF-8:CS/PES composite membrane with CO_2_ permeance of 26.6 GPU and a selectivity of 24.2 [40], and a CNT:PVAm-PVA/PSf composite membrane with a CO_2_ permeance of 129 GPU and a CO_2_/CH_4_ selectivity of 45 [30]. The simulations were run by varying the stage-cut in the range from 0.1 to 0.9 and the results are shown in Figure 10 and Figure 11 for CO_2_ and CH_4_, respectively.

Firstly, Figure 10 considers the results obtained at the permeate side, for CO_2_, observing the process performance, since an increase in CO_2_ recovery implies a decrease in CO_2_ purity, as more CO_2_ goes through the membrane to the permeate but the permeate quality is lower with increasing stage-cut. The PVAm-PVA based membrane provides a purity of 89%, followed by the grafted ZIF-8:CS composite membrane, with a purity of 82.5% and the CA hollow fiber membrane a CO_2_ purity of 69%, which is closer to the membranes prepared in this work. On the other hand, all the membranes analyzed reached a CO_2_ recovery higher than 99%, at high values of stage-cut. These results provide evidence of the possibility of using green alternative materials to fabricate membranes with similar performance as other membranes.

Figure 11 shows the trade-off for CH_4_ purity and recovery in the retentate. As before, when purity increases, recovery decreases, but in this case, the results provided for our membranes are in the same order of magnitude as those of the literature membranes. IL-CS and AM-4:IL-CS composite membranes could achieve a purity of 97.2%, very close to the literature ones.

## 4. Conclusions

Membrane technology is considered as a sustainable technology for biogas purification, but the membrane materials used for the fabrication of commercial membranes are still based on fossil fuels and costly reactants and solvents, thus biopolymer-based membranes are being studied as a green alternative for CO_2_/CH_4_ separation. In this work, composite membranes made of chitosan matrix hybridized by [emim][acetate] IL and different nano-porous fillers previously studied in our laboratory: CO_2_-sorptive AM-4 layered titanosilicate, nanoporous UZAR-S3 lamellar stannosilicate and nanometric sized HKUST-1. The CO_2_/CH_4_ performance of the mixed matrix composite membranes was evaluated experimentally in single gas and gas mixture CO_2_/CH_4_ mode separation. Although the permeance and selectivity are still below commercial membranes and further work is being carried out to increase performance of these chitosan-based membranes, the hydrophilic and compatibility factor of the membrane components seem to play a significant role in facilitating the transport of CO_2_ across the membrane and influencing the purity and recovery of both gases in the permeate and retentate, respectively.

Besides, in this work a mathematical model was applied to the CO_2_/CH_4_ separation with different types of bio-based membranes. The model was first validated using a commercial PDMS membrane and then the chitosan-based composite membrane performance was analyzed in terms of the target objectives of CO_2_ and CH_4_ recovery and purity in the permeate and retentate, respectively. The IL-CS and AM-4:IL-CS composite membranes, of higher hydrophilic and CO_2_ adsorptive character, showed the most promising results, close or even surpassing those of the hydrophobic commercial membrane used as reference. This provides scope for alternative membrane materials fabricated from renewable or bio-degradable polymers and non-toxic fillers to show at least comparable CO_2_/CH_4_ separation as existing membranes, as well as the simultaneous feedback on membrane development enabled by the simultaneous correlation of the process requirements with the membrane properties to achieve those process targets by the simulation and optimization tools supporting the experimental work.

## Figures and Tables

**Table 1:** Water Uptake and Swelling Degree calculation from measurements of dry and wet thickness and weight for the composite membranes studied in this work.

Membrane	Thickness (µm)		Weight (g)		WU (%)	SD (%)
	Dry	Wet	Dry	Wet		
PES	132		0.072			
IL:CS/PES (PDMS) ^a^	151 ± 10	215 ± 50	0.225 ± 0.06	0.349 ± 0.052	55 ± 17	42 ± 27
IL:CS/PES (TMC) ^b^	144 ± 4.4	237 ± 42	0.229 ± 0.028	0.341 ± 0.047	49 ± 12	64 ± 28
AM-4:IL-CS/PES	173 ± 22	222 ± 15	0.137 ± 0.004	0.223 ± 0.015	63 ± 9.4	28 ± 9.1
UZAR-S3:IL-CS/PES	174 ± 0.1	194 ± 4.9	0.136 ± 0.002	0.209 ± 0.007	54 ± 2.8	11 ± 2.4
HKUST-1:IL-CS/PES	180 ± 2.8	214 ± 6.3	0.134 ± 0.007	0.256 ± 0.006	90 ± 5.3	19 ± 1.7

^a^ IL:CS coating prepared over a PDMS gutter layer over the microporous PES support; ^b^ IL:CS coating prepared by the modified interfacial polymerization method as reported elsewhere [46].

## Data Availability

Not applicable.

## Nomenclature

Symbol	Description
AM-4	Layered titanosilicate
A	Membrane area (cm^2^)
Barrer	Unit of permeability (1 barrer = 10^−10^ cm^3^ (STP) cm^−1^ s^−1^ cmHg^−1^)
CA	Cellulose acetate
CH_4_	Methane
CNC	Crystalline nanocellulose
CNTs	Carbon nanotubes
CO_2_	Carbon dioxide
CS	Chitosan
[emim][Ac]	1-ethyl-3-methylimidazolium [emim][Ac]
*F_r,k_*	Total molar flowrate of retentate (kmol h^−1^) in cell k
*F_p,k_*	Total molar flowrate of permeate (kmol h^−1^) in cell k
GPU	Unit of permeance (1 GPU = 10^−6^ cm^3^ (STP) cm^−2^ s^−1^ cmHg^−1^)
HKUST-1	Metal organic framework (MOF)
IL	Ionic liquid
***l***	Selective layer thickness for the separation (mm)
MMM	Mixed matrix membrane
*N_comp,k_*	Molar flowrate of each component permeating though the membrane cell k (kmol h^−1^)
NaOH	Sodium hydroxide
Na_2_SiO_3_	Sodium silicate
Nps	Nanoparticles
*P*	Permeability of the selective membrane layer (Barrer)
*p_r_*	Retentate pressure (bar)
*p_p_*	Permeate pressure (bar)
PDMS	Polydimethyl siloxane
PEG	Polyethylene glycol
PES	Polyethersulfone
PSf	Polysulfone
PU	Polyurethane
PVA	Polyvinyl alcohol
PVAm	Polyvinyl amine
*Q_p_*	Permeate flow rate (cm^3^ (STP) s-1)
SnCl_2_ ·2H_2_O	Tin(II) chloride di-hydrated
TiO_2_	Anatase
TMC	Trimesoyl chloride
UZAR-S3	Layered stannosilicate
*α_i/j_*	Selectivity of component i over j
*x_comp,k_*	Molar fraction of each component in the permeate
*y_comp,k_*	Molar fraction of each component in the retentate

## Appendix A

**Table A1:** Comparison of selected biopolymer-based membranes for CO_2_/CH_4_ separation with the membranes evaluated in this work.

Membrane) ^1^	Selective Layer Thickness [μm]	CO_2_ Permeability [Barrer]	CO_2_/CH_4_ Selectivity [–]	Reference
CA (18 wt%) HF	50	1.26	7.9	[66]
CNT (1 wt%)/CA (3 wt%)	35	14.2	21.2	[67]
NH_2_-MIL-53(Al) (15 wt%)/CA (10 wt%)		52.6	28.7	[68]
PVAm-PVA blend	0.6	31.2	30	[29,65]
CNC/PVA	0.8	86	43	[31]
CNT (1 wt%)/PVAm-PVA	1.5	129	45	[30]
NiO (5 wt%)/PU (10 wt%)	100	321	21.76	[32]
IL-PEG-PU	97.5	499	44	[33]
SAPO-34 (20 wt%)/PU (3 wt%)	65	28.71	25.63	[35]
CS-gC_3_N_4_-ZIF-8/PES	20	180	24.2	[40]
IL-CS/PES	14	1024	16	[47]
HKUST-1(5 wt%)-IL-CS/PES	67	26,872	30	[47]
IL-CS/PES	22.4	154 ± 18	4.26	This work
AM-4:IL-CS/PES	52.5	287 ± 132	4.25	This work
UZAR-S3:IL-CS/PES	42.5	129 ± 19	1.88	This work
HKUST-1-IL-CS/PES	50.4	167 ± 32	2.21	This work

^1^ PU: polyurethane; PVAm: polyvinyl acetylamide; PVA: polyvinyl alcohol; CS: chitosan; gC_3_N_4_ grafted carbonnitrile; pes: polyether sulfone support; CNT: carbon nanotubes; CA cellulose acetate; CNC: semicrystals of nanocellulose.

**Table A2:** Experimental CO_2_ permeate purity and recovery as a function of CO_2_ concentration in the feed and experimental stage-cut for the commercial PDMS membrane. The error of the model validation calculated by Equation (14) is given in the last columns.

CO_2_ in Feed (v%)	Stage-Cut	CO_2_ Purity in Permeate (%)	CO_2_ Recovery (%)	AARE_purity_ (%)	AARE_recovery_ (%)
20	0.30	26.67 ± 0.5	39.98 ± 6.08	2.60	2.67
35	0.31	42.55 ± 0.36	50.60 ± 2.76	16.02	13.59
50	0.46	57.81 ± 0.52	52.99 ± 1.80	14.63	15.06
70	0.59	74.24 ± 0.24	62.76 ± 1.82	8.62	8.31

**Table A3:** Experimental CH_4_ permeate purity and recovery as a function of CH_4_ concentration in the feed and experimental stage-cut for the commercial PDMS membrane. The error of the model validation calculated by Equation (14) is given in the last columns.

CH_4_ in Feed (v%)	Stage-Cut	CH_4_ Purity in Permeate (%)	CH_4_ Recovery (%)	AARE_purity_ (%)	AARE_recovery_ (%)
30	0.59	25.75 ± 0.28	50.79 ± 1.47	24.84	25.05
50	0.46	42.19 ± 0.52	38.68 ± 1.65	20.05	19.77
65	0.31	57.44 ± 0.36	27.25 ± 1.49	11.87	12.86
80	0.30	72.64 ± 0.27	30.49 ± 4.39	0.95	10.67

**Table A4:** Experimental CO_2_ retentate purity and recovery as a function of CO_2_ concentration in the feed and experimental stage-cut for the commercial PDMS membrane. The error of the model validation calculated by Equation (14) is given in the last columns.

CO_2_ in Feed (v%)	Stage-Cut	CO_2_ Purity in Retentate (%)	CO_2_ Recovery (%)	AARE_purity_ (%)	AARE_recovery_ (%)
20	0.30	17.11 ± 0.63	60.02 ± 6.10	1.82	1.99
35	0.31	31.63 ± 0.31	62.51 ± 2.04	9.54	9.77
50	0.46	43.39 ± 0.50	47.01 ± 1.81	16.71	16.97
70	0.59	63.83 ± 0.24	37.24 ± 1.82	14.46	14.13

**Table A5:** Experimental CH_4_ retentate purity and recovery as a function of CH_4_ concentration in the feed and experimental stage-cut for the commercial PDMS membrane. The error of the model validation calculated by Equation (14) is given in the last columns.

CH_4_ in Feed (v%)	Stage-Cut	CH_4_ Purity in Retentate (%)	CH_4_ Recovery (%)	AARE_purity_ (%)	AARE_recovery_ (%)
30	0.59	36.17 ± 0.62	49.21 ± 1.47	25.52	26.07
50	0.46	56.61 ± 0.50	61.32 ± 1.65	12.81	12.47
65	0.31	68.37 ± 0.30	72.75 ± 1.49	4.41	4.00
80	0.30	82.88 ± 0.63	72.49 ± 4.39	0.38	0.41
